# Psychobiological responses to choir singing and creative arts activities in children and adolescents with mental disorders: results of a pilot study

**DOI:** 10.1007/s40211-024-00502-6

**Published:** 2024-07-25

**Authors:** Katarzyna Grebosz-Haring, Leonhard Thun-Hohenstein

**Affiliations:** 1 Interuniversity Organisation Science & Arts, Paris Lodron University Salzburg, Mozarteum University Salzburg, Salzburg, Austria; 2 Department of Art History, Musicology and Dance Studies, Paris Lodron University Salzburg, Salzburg, Austria; 3 Salzburg Institute for Arts and Medicine (SIAM), Salzburg, Austria; 4 Paracelsus Medical University, Salzburg, Austria

**Keywords:** Child and adolescent psychiatry, Arts interventions, Stress, Salivary cortisol, Immunoglobulin A, Kinder- und Jugendpsychiatrie, Kunstinterventionen, Stress, Kortisol im Speichel, Immunglobulin A

## Abstract

**Background:**

Children and adolescents living with mental health problems often experience stress and poor mood states, which may influence their quality of life and well-being. Arts interventions may improve mood and well-being and reduce physiological stress in this vulnerable population.

**Methods:**

A cohort of patients in child and adolescent psychiatry (*N* = 42; age range: 12–18 years) participated in one of four arts activities including choir singing (*n* = 11), textile design (*n* = 9), drama (*n* = 16), and clownery (*n* = 6). They were led by professional artists and delivered through five consecutive 90-min daily afternoon sessions over the course of 1 week. Questionnaires of mood and saliva samples before and after each session served to assess short-term psychobiological changes. In addition, patients reported their quality of life and well-being at the beginning and at the end of the 1‑week program.

**Results:**

Results showed that *alertness* was significantly enhanced after textile design (∆post–pre = 4.08, 95% CI [0.77, 7.39]) and after singing (∆post–pre = 2.20, 95% CI [−0.55, 4.94]). Moreover, *mood* tended to be positively affected by textile design (∆post–pre = 2.89, 95% CI [−0.39, 6.18]). Quality of life increased significantly after singing (∆post–pre = 5.49, 95% CI [1.05, 9.92]). Arts participation except singing was associated with significant reductions in salivary cortisol (sCort) (textile design ∆post–pre = −0.81 ng/mL, 95% CI [−1.48, −0.14]; drama ∆post–pre = −0.76 ng/mL, 95% CI [−1.28, −0.24]; clownery ∆post–pre = −0.74 ng/mL, 95% CI [−1.47, −0.01]). No significant changes were observed for well-being over the whole program and salivary immunoglobulin A (sIgA) after any of the arts activities.

**Discussion:**

These results suggest that arts participation can improve mood state and reduce stress in young people with mental disorders, but there is a need for further studies.

## Abstract (de)

**Hintergrund:**

Kinder und Jugendliche mit psychischen Problemen leiden häufig unter Stress und negativer Stimmung, was ihre Lebensqualität und ihr Wohlbefinden beeinträchtigen kann. Künstlerische Interventionen können die Stimmung und das Wohlbefinden verbessern und den physiologischen Stress in dieser gefährdeten Bevölkerungsgruppe reduzieren.

**Methodik:**

Eine Kohorte von Patienten der Kinder- und Jugendpsychiatrie (*N* = 42; Altersspanne: 12–18 Jahre) nahm an einer von 4 künstlerischen Aktivitäten teil, darunter Chorgesang (*n* = 11), Textildesign (*n* = 9), Schauspiel (*n* = 16) und Clownerie (*n* = 6). Diese wurden von professionellen Künstler*innen geleitet und über einen Zeitraum von einer Woche in fünf 90-minütigen, täglichen Nachmittagssitzungen durchgeführt. Fragebögen von Stimmungs- und Speichelproben vor und nach jeder Sitzung dienten dazu, kurzfristige psychobiologische Veränderungen zu erfassen. Darüber hinaus berichteten die Patienten zu Beginn und am Ende des einwöchigen Programms über ihre Lebensqualität und ihr Wohlbefinden.

**Ergebnisse:**

Die Ergebnisse zeigten, dass die *Wachsamkeit *nach Textildesign (∆post-prä = 4,08, 95%-KI [0,77, 7,39] und Singen (∆post-prä = 2,20, 95%-KI [−0,55, 4,94] signifikant erhöht war. Darüber hinaus wurde die *Stimmung *tendenziell durch Textildesign positiv beeinflusst (∆post-prä = 2,89, 95%-KI [−0,39, 6,18]). Die Lebensqualität stieg nach dem Singen signifikant an (∆post-prä = 5,49, 95%-KI [1,05, 9,92]). Die Teilnahme an den künstlerischen Aktivitäten mit Ausnahme des Singens war mit einer signifikanten Reduktion des Kortisolspiegels verbunden (Textildesign ∆post-prä = −0,81 ng/ml, 95%-KI [−1,48, −0,14]; Schauspiel ∆post-prä = −0,76 ng/ml, 95%-KI [−1,28, −0,24]; Clownerie ∆post-prä = −0,74 ng/ml, 95%-KI [−1,47, −0,01]). Es wurden keine signifikanten Veränderungen des Wohlbefindens während des gesamten Programms und des Immunglobulins A nach einer der künstlerischen Aktivitäten beobachtet.

**Schlussfolgerung:**

Diese Ergebnisse weisen deutlich daraufhin, dass die Teilnahme der psychisch kranken Kinder und Jugendlichen an künstlerischen Aktivitäten deren Stimmung verbessern und den Stress senken kann. Gleichzeitig zeigt sich auch die Notwendigkeit weiterer Studien.

## Introduction

Mental disorders (MDs) in children and adolescents are highly prevalent, even in high-income countries [1]. The prevalence increased due to the global impact of COVID-19 on MDs in this population [2]. The pathogenesis of MDs is thought to be a complex process influenced by individual and environment factors [3]. Generally, MDs affect young people’s emotional state (mood; [4]) and are characterized by atypical perceptions, behavior, and relationships [5]. This, in turn, limits young people’s participation in age-appropriate activities, quality of life, and overall well-being [5]. Moreover, young people with MDs often lose access to their creativity and spontaneity, experiencing despair in the face of daily challenges [6] and heightened levels of stress [7, 8].

In general, stress is associated with multifaceted behavioral and biological responses, primarily by activating the hypothalamus–pituitary–adrenal (HPA) axis [9]. The secretion of cortisol occurs under the influence of the circadian rhythm in the adaptation process to environmental challenges. Thus, salivary sCort is a correlate of the biological stress response and is essential to maintain homeostasis [9]. Over the course of the day, the highest level is reached in the 30–45 min after waking up, the lowest is reached around midnight. The early afternoon is an appropriate period for evaluating potential alterations in the activity of the HPA axis, triggered, for instance, by arts-related activities [10]. Stress may cause alterations in immune function [11]. Immunoglobulin A, a first-line mucosal protector against pathogens [11], is produced in response to physical and psychological stress. It is also affected by an individual’s emotional state [12].

The causal connection between MDs and stress appears bidirectional. For example, biographical stress experiences in early life may contribute to MDs [13]. However, the development of MDs is also associated with the dysregulation of neuroendocrine system activities and underlying stress responses [14]. Therefore, it is likely that both genetic components and gene–environment interactions give rise to MDs from an early age [15].

Previous studies have addressed sCort and sIgA alongside other biomarkers in the context of arts participation, for example, with respect to amateur adult singers [16, 17]. Findings suggest that favorable short-term psychobiological changes can occur in response to such interventions, but there is little information with respect to vulnerable groups including children and adolescents with MDs [18, 19].

Creative arts therapies and interventions are common strategies in child and adolescent psychiatry (e.g., [20–22]). However, the empirical evidence appears mixed. For example, cognitive behavioral therapy can be more effective than arts or music therapy for reducing symptoms of depression in children [23]. By contrast, a meta-analysis of 11 experimental studies on music therapy found a moderate positive effect on clinical outcomes but identified a need for more studies in clinical settings [24]. Finally, participation in activities such as listening to and making music can elicit positive feelings and influence stress symptoms in young people with mental health issues [18]. Creative arts engagement can reduce anxiety and depression symptoms in young people [25], while enhancing self-confidence and self-esteem, a sense of achievement and empowerment, social skills, positive behavioral changes, and resilience [26–28]. However, a systematic review of adjuvant interventions suggests that creative arts participation appears to be beneficial but, overall, less effective than sports and yoga [29].

It is of note that the term “therapeutic” typically implies the involvement of a trained therapist, while delivery through care staff or external providers suggests an emphasis on leisure or distraction activity [28]. However, beneficial effects can occur, irrespective of the specific therapeutic goal settings. For example, Grebosz-Haring and Thun-Hohenstein [6, 18] argue that engagement in arts activities can stimulate creative processes to increase self-confidence and self-efficacy, and bring distraction, attention, imagery, joy, and pleasure. The arts can also encourage young people to engage in more positive self-reflections and social interactions.

Creative arts interventions for hospitalized children and adolescents with diagnosed MDs produced mixed findings (e.g., [18, 22]), probably due to conceptual and methodological flaws, which question the validity of conclusions (e.g., [30]). Therefore, it is unclear, how different types of arts activities (musical vs. non-musical) affect young people with MDs in a clinical setting. Further, there is also a need to study the feasibility and treatment effects with respect to the individual needs and paths of recovery and rehabilitation in this vulnerable group.

### The present study

The present study is part of a 2-year pilot project to assess the feasibility of a music- and arts-based intervention protocol for hospitalized children and adolescents with MDs. Specifically, we were interested in the effectiveness of short-term intensive music and arts activities on behavioral measures of mood, quality of life, and well-being as well as on biological markers related to stress and immune function. These interventions included choir singing, textile design, drama, and clownery, which were incorporated into standard treatment routines. Results of specific subsets of the protocol concerning music-related interventions were published elsewhere [18].

We asked the following research question:

#### RQ1:

What are the effects of short-term arts-based interventions for young people with MD on (a) behavioral measures of mood, quality of life, and well-being as well as on (b) the concentrations of sCort and sIgA in a clinical setting?

We expected that the music and creative arts activities would induce positive changes in psychological outcomes such as improvement of self-reported mood state, quality of life, and psychological well-being (H1). Furthermore, we also assumed positive changes in neuroendocrine stress (HPA axis) and immune function in terms of a reduction in sCort and an increase in sIgA (H2).

A subsidiary goal of the study was to investigate the compliance, appropriateness of inclusion criteria, attrition, dropout rate, and possible factors that might facilitate or compromise participation in clinic-based arts intervention. It is of note that individual mental conditions could interact with preferences in the different types of arts activities. Therefore, it is important to consider individual accounts of participation. However, these data are subject to a separate investigation and will not be part of the present paper. Finally, we sought to reflect on the acceptability of gathering biomarker data during a clinical intervention in the clinical setting.

## Methods

### Procedure and participants

The study was a prospective, parallel four-group pilot trial that compared different forms of creative arts activities (musical and non-musical): group singing, textile design, drama, and clownery. The sample included young patients in inpatient or day-clinic treatment at the University Department for Child and Adolescent Psychiatry. Recruitment at the hospital took place from 14 days to 3 days before the beginning of the interventions. Patients were eligible if they were 10–18 years old with a current diagnosis of mental disorder, defined according to Chapter V (F00–99) of ICD-10. They were included regardless of their medication status and any concurrent therapies and received treatment as usual in parallel with the artistic interventions. Musical or artistic skills were not required. Exclusion criteria were diagnoses with any significant hearing impairments, acute state of confusion, inability to verbalize, suicidal tendency, addictive behavior, physical threat of self-harm or aggression, and previous participation in the same intervention. Following the orientation of other studies (e.g., [17, 31]), the target sample size of 20 patients per intervention was considered adequate, although no formal sample size calculation was performed.

Two singing, textile design, drama, and one clownery intervention were conducted. Patients could not choose the type of intervention but were assigned to the intervention type that was available at the time they were admitted to the clinic.

All interventions took place in a group setting and consisted of 90-min sessions occurring daily from Monday through Friday. The feasibility of conducting 1‑week daily sessions was facilitated by the clinical setting structured daily routines and the patients’ duration of stay. Due to circadian variations in salivary cortisol levels, each session was carried out in the afternoon between 2:00 and 3:30 pm. At baseline (3 days before the sessions started), patients completed questionnaires on sociodemographic variables. Child psychiatrists recorded data regarding psychiatric conditions and medication for each patient. The study protocol was approved by the Salzburg State Ethics Committee with the reference number 415-E/1787/4-2014. Written informed consent from each patient, parent, or legal guardian was obtained prior to the study.

### Interventions

All arts interventions were led by experienced artists without therapeutic background in four groups specialized for one art intervention: singing, textile design, drama, and clownery. Singing sessions initially focused on an approximately 10-min-long breathing and vocalization phase. For the rest of the session, songs chosen by the conductor as well as songs known by the participants were rehearsed (see repertoire pieces in [18]). The creative process during the workshops encompassed activities such as painting, sewing, printing, and the use of elements like color, form, expression, voice, and body movement. Textiles and theatrical play were the mediums through which the forces of self-determination and confidence-building creativity were channeled. All workshops were focused on the creation of a performance centered around the theme of “Settling Down,” with the goal of developing a concluding theatrical performance, a film production, and an art exhibition. Please see Table 1 for a brief description of each intervention type summarizing the core activities.

**Table 1:** Arts description

Art form	Warm-up	Duration	Art intervention	Material	Total duration
Singing	Breathing and voice training	10 min	Choir singing	Prepared repertoire plus chosen by kids	90 min
Textile design	Planning and preparation of the session	10–15 min	Designing, sketching, cutting, sewing; preparation of the chairs	Sewing material; fabrics of different kind; woods; different chairs	90 min
Theater	Drama pedagogic warm-up	10–15 min	Writing, storytelling, improvisation, acting and drama production	Paper, pencil	90 min
Clownery	Short warm-up clownery by clowns	5 min	Cooperative planning and working out of the workshop; improvisation and group performance	Cloths, hats, noses	90 min

### Measures

#### Assessment of subjective current mood state

Mood state was assessed using the Multidimensional Mood Questionnaire (*Der Mehrdimensionale Befindlichkeitsfragebogen* [MDBF]), a well-validated tool for screening current mood state that is mainly used in clinical practice and research [32]. The MDBF contains three mood dimensions (good—bad mood [GM], alertness—tiredness [AT], and calmness—restlessness [CN]) made up of 24 items in total. Each of the three scales includes eight items rated on a five-point Likert scale, with the items for each subscale added together. A higher score means *positive mood, alertness*, and *calmness*. The questionnaire was given to the patients daily before and after each 90-min singing or arts intervention.

#### Psychological measurements of health-related quality of life and psychological well-being

Health-Related Quality of Life (HRQL) was assessed using the Pediatric Quality of Life Inventory (PedsQL; [33]), which is a brief measure for children and young people. It consists of 23 items using a five-point Likert scale. There are four subscales: Physical Functioning (eight items), Emotional Functioning (five items), Social Functioning (five items), and School Functioning (five items). The sum of the four scales generates an overall score of HRQL.

Well-being was assessed using the Warwick–Edinburgh Mental Wellbeing Scale (WEMWBS; [34, 35]). The WEMWBS is suitable for participants aged 16 and above as well as for use at a population level in teenagers aged 13 and above. Measurements were taken at two time points: at baseline (3 days before the sessions started) and post-intervention (after the last session). Higher scores reflect greater HRQL and well-being.

#### Biological measurements of momentary stress and immune function

Stress was assessed using sCort, as it is an objective measure for stress level (short-term neuroendocrine stress; HPA axis), and immune function was assessed using sIgA (as a central measure for short-term immune function). Saliva samples were collected immediately before and after each 90-min arts intervention using the Salivette® tube from Sarstedt. Patients were asked to chew a cotton suction ball for about 1 min. After each sampling, this swab was placed back into the tube and cooled in the refrigerator at 4 °C for about 1.5 h before being stored at −20 °C until it was analyzed. Saliva analyses were conducted at the central laboratory of the Christian Doppler Clinic/PMU, Salzburg, using an electrochemiluminescent-immunoassay (Cobas®-6000) for sCort and a nephelometric measuring method for sIgA. sCort is reported in ng/mL and sIgA in mg/dL.

### Statistical analysis

Standard descriptive statistic measures were calculated for all variables. Confidence intervals for MDBF, sCort, and sIgA were calculated for the mean differences (postintervention–preintervention [post–pre]) over all of the days using mixed model analysis in SPSS. All available data were included (irrespective of the number of days an individual had participated) and the dependence between repeated measurements was modeled using the compound symmetry correlation matrix (for all participants on all days, the differences between the post–pre measurements have a common variance σ^2^ and measurements on different days are correlated by a fixed correlation ϱ). For PedsQL and WEMWBS, we calculated the means, standard deviations, standard errors, and 95% confidence intervals for the post–pre differences (final measurement—baseline measurement) using standard methods (based on the *t* distribution). We did not calculate *t* values because the sample sizes are too small. The statistical data analysis was conducted using IBM® SPSS® Statistics, Version 18.0.

## Results

### Participants

**Fig. 1:**
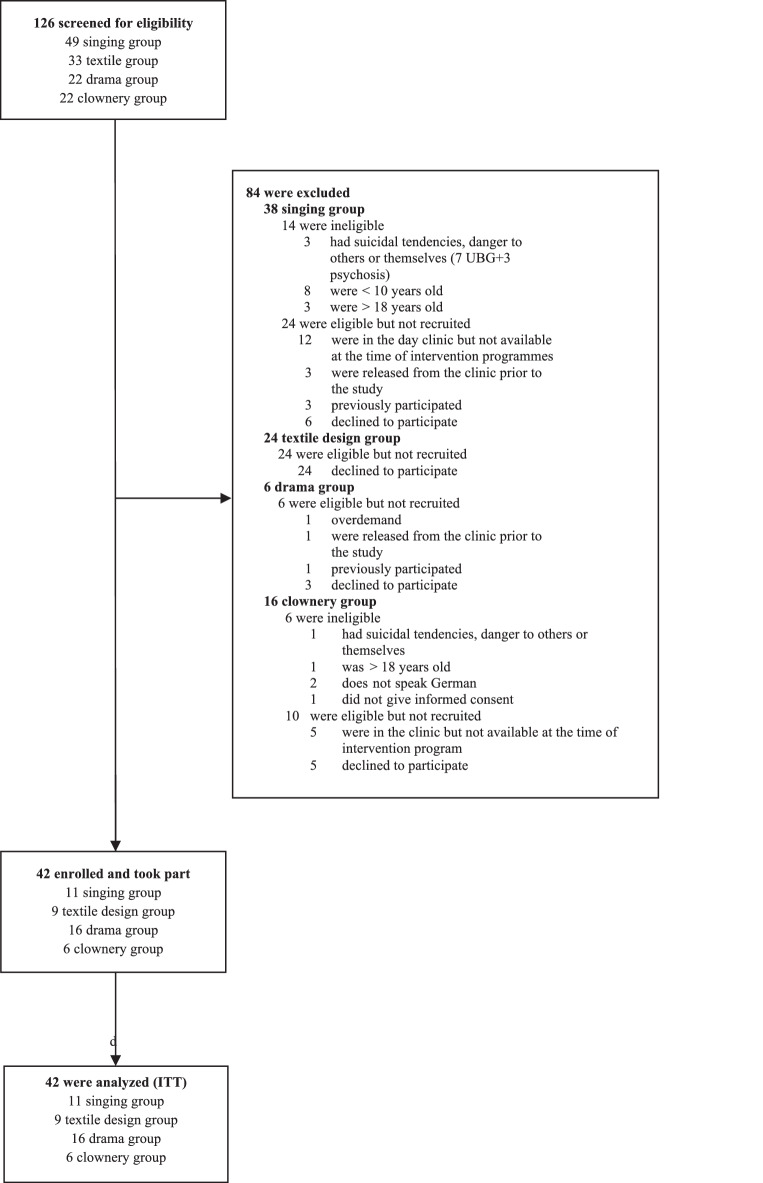
Consort-type flow diagram

In total, 126 patients were screened for eligibility, of whom 42 (33.3%) were enrolled for four conditions (choir singing *n* = 11, textile design *n* = 9, drama *n* = 16, and clownery *n* = 6). The sample sizes were smaller than expected because the number of participants who were not eligible and/or did not want to participate was higher than initially anticipated. Thus, it may be assumed that the inclusion criteria were too restrictive. Nevertheless, given the severity of mental health issues in this population, the overall participation was sufficient to ensure that the different interventions also allowed for social interactions within each group, which is seen as part of the rehabilitation process. All 42 enrolled patients entered interventions and all patients who participated were included in the analysis (Fig. 1).

The samples were closely equivalent across the four arms (see Table 2). The following noticeable differences were observed: the age range of patients in the clownery group was lower than in the other groups. Moreover, the number of patients with medication was higher in the arts groups compared with the singing group.

**Table 2:** Sample characteristics by intervention arm

	Singing (*n* = 11)	Textile design (*n* = 9)	Theater (*n* = 16)	Clownery (*n* = 6)
*Age (years), mean (SD)*	15.6 (1.6)	16.0 (1.6)	15.9 (1.6)	13.8 (1.7)
*Age range, years*	13–18	13–18	13–19	12–16
*Sex, male, n*	3	4	6	0
*School type, n*	(*n* = 10)	(*n* = 6)	(*n* = 11)	(*n* = 4)
Lower secondary education	2	3	1	3
Trade school	2	1	4	0
Gymnasium (secondary school)	5	2	4	1
University/academy/college	1	0	1	0
Special school	0	0	1	0
*Diagnosis of psychiatric disorders (ICD-10), n*	(*n* = 11)	(*n* = 9)	(*n* = 13)	(*n* = 5)
Affective disorders (F3)	2	1	3	1
Neurotic and posttraumatic disorders (F4)	3	3	2	4
Eating disorders (F5)	3	0	4	0
Personality disorders (F6)	1	1	0	0
Autism (F8)	0	0	0	0
Attention deficit disorders (F9)	0	2	3	0
Mix of disorders	2	2	1	0
*Medication, n*	(*n* = 11)	(*n* = 9)	(*n* = 13)	(*n* = 5)
Neuroleptics	2	2	2	2
Stimulants	0	0	1	0
Sedatives	0	1	1	0
Antidepressants	0	1	2	1
Vitamin supplements	0	0	1	0
No medication	9	3	4	2
Multiple medications	0	1	2	0

### Subjective current mood state

**Fig. 2:**
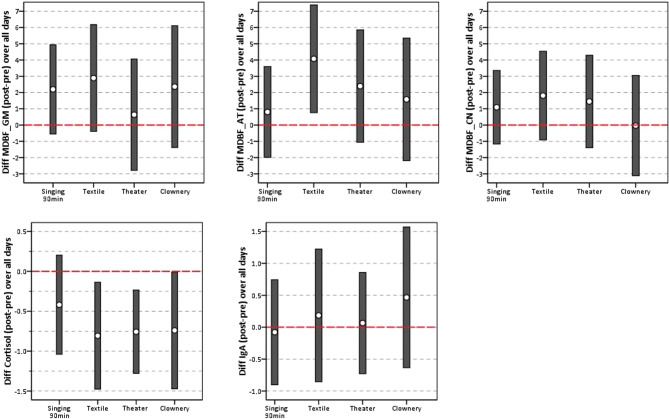
95% confidence intervals for differences (post-pre) in MDBF subscales (GM, AT, CN), sCort, and sIgA by intervention arm

Over all of the days, a significant pre–post change in the dimension *alertness *(MDBF_AT; mean 4.08, 95% CI [0.77, 7.39]) was observed in the textile design intervention. Moreover, a tendency toward a positive pre–post change in the dimension *mood* (MDBF_GM) was observed in the singing intervention (mean 2.20, 95% CI [−0.55, 4.94]) and in the textile design intervention (mean 2.89, 95% CI [−0.39, 6.18]). Table 3 and Fig. 2 display the 95% confidence intervals for differences (post–pre) in psychological and biological outcomes by intervention arm over all of the days.

**Table 3:** Means, standard errors of the means (SEs), and 95% confidence intervals for mean differences (post-pre) in MDBF subscales (GM, AT, CN), sCort, and sIgA by intervention arm over all days

				95% CI	
	Art activity	Mean	SE	Lo	Hi
Diff. MDBF_GM (post–pre)	Singing	2.20	1.34	−0.55	4.94
	Textile	2.89	1.62	−0.39	6.18
	Theater	0.64	1.68	−2.79	4.07
	Clownery	2.36	1.82	−1.38	6.11
Diff. MDBF_AT (post–pre)	Singing	0.81	1.35	−1.99	3.60
	Textile	4.08	1.62	0.77	7.39
	Theater	2.39	1.68	−1.06	5.85
	Clownery	1.58	1.82	−2.19	5.35
Diff. MDBF_CN (post–pre)	Singing	1.10	1.10	−1.16	3.36
	Textile	1.81	1.34	−0.91	4.54
	Theater	1.45	1.39	−1.39	4.29
	Clownery	−0.03	1.50	−3.12	3.05
Diff. sCort ng/mL (post–pre)	Singing	−0.42	0.30	−1.04	0.20
	Textile	−0.81	0.33	−1.48	−0.14
	Theater	−0.76	0.25	−1.28	−0.24
	Clownery	−0.74	0.34	−1.47	−0.01
Diff. sIgA mg/dL (post–pre)	Singing	−0.08	0.40	−0.90	0.74
	Textile	0.18	0.51	−0.85	1.22
	Theater	0.07	0.39	−0.73	0.86
	Clownery	0.47	0.53	−0.63	1.57

*GM *good—bad mood, *AT* alertness—tiredness, *CN* calmness—restlessness

As can be seen from Fig. 2, only a few confidence intervals show a significant difference between pre and post values (since in most cases the “0” is included in the confidence intervals). The mean value differences in sCort (textile design/drama/clownery) and MDBF_AT (textile design) indicate a significant pre–post change. For MDBF_GM, the confidence intervals for singing and textile design are only slightly below 0; here, there is a tendency toward a positive change (the 90% CIs would probably lie above the 0 line).

### Health-related quality of life and psychological well-being

Initially, the baseline WEMWBS score for well-being tended to be lower in the clownery group than in the other groups (the baseline statistics for PedsQL and WEMWBS are given in Table 4).

**Table 4:** Baseline data for PedsQL and WEMWBS (mean/SD)

Psychological scale	Singing	Textile design	Theater	Clownery
–	(*n* = 10)	(*n* = 7)	(*n* = 9)	(*n* = 6)
PedsQL, mean (SD)	71.06 (11.68)	61.12 (10.44)	65.27 (19.12)	58.61 (14.14)
–	(*n* = 10)	(*n* = 7)	(*n* = 14)	(*n* = 6)
WEMWBS, mean (SD)	42.2 (7.48)	40.43 (5.56)	41.43 (10.93)	25.17 (7.22)

*PedsQL* Pediatric Quality of Life Inventory™, *WEMWBS* Warwick–Edinburgh Mental Wellbeing Scale

**Fig. 3:**
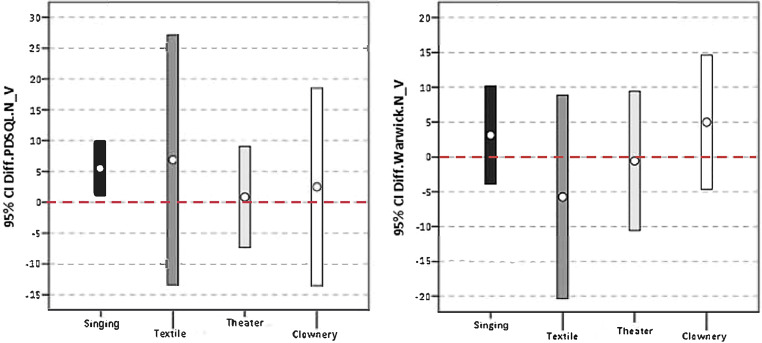
Means and 95% confidence intervals for PedsQL and WEMWBS by intervention arm (post-pre)

The means and 95% confidence intervals for patient-reported HRQL and well-being are shown in Table 5 and Fig. 3. The HRQL increased significantly in the singing intervention (5.49, 95% CI [1.05, 9.92]), but not in the other arts interventions. There were no significant changes observed in well-being.

**Table 5:** Means, standard errors of the means (SEs), and 95% confidence intervals for mean differences (post–pre) in PedsQL and WEMWBS by intervention arm

				95% CI	
	Art activity	Mean	SE	Lo	Hi
Diff. PedsQL (post–pre)	Singing	5.48	2.85	1.051	9.92
	Textile	6.88	1.46	−13.38	27.15
	Theater	0.84	0.24	−7.37	9.06
	Clownery	2.50	0.43	−13.52	18.52
Diff. WEMWBS (post–pre)	Singing	3.13	1.05	−3.92	10.17
	Textile	−5.75	−1.25	−20.35	8.85
	Theater	−0.57	−0.14	−10.56	9.42
	Clownery	5.00	1.65	−4.63	14.63

*PedsQL* Pediatric Quality of Life Inventory™, *WEMWBS* Warwick–Edinburgh Mental Wellbeing Scale

### Salivary cortisol and immunoglobulin-A

A significant mean drop in sCort was observed in the arts interventions (textile design mean −0.81, 95% CI [−1.48, −0.14]; drama mean −0.76, 95% CI [−1.28, −0.24]; clownery mean −0.74, 95% CI [−1.47, −0.01]). There was no significant difference after the singing intervention. Moreover, no significant pre–post differences in the level of sIgA were found (Table 3; Fig. 2).

Performing biological measurements with salivary cortisol has been shown to be a feasible and noninvasive method in the clinical setting of children and adolescents with mental illnesses.

### Intervention adherence

Of the 42 participants included, 17 participants (41%) attended on ≤ 3 days, 11 participants (26%) attended on 4 days, and 14 participants (33%) attended on all 5 days. The reasons for the low level of participation are, for example, illness or other activities at the clinic such as school attendance. Adherence varied between groups as well, with the singing group having a higher attendance rate (55% attended 5 days) than the other groups (textile and clownery 33%, theater 19% attended 5 days; for details on case numbers per day and intervention, see Table 6).

**Table 6:** Case numbers per day and intervention

Attendance	Singing (*n* = 11)		Textile design (*n* = 9)		Theater (*n* = 16)		Clownery (*n* = 6)	
	*N*	%	*N*	%	*N*	%	*N*	%
1 day	1	9.1	2	22.2	5	31.3	1	16.7
2 days	0	0	2	22.2	2	12.5	0	0
3 days	2	18.2	1	11.1	1	6.3	0	0
4 days	2	18.2	1	11.1	5	31.3	3	50.0
5 days	6	54.5	3	33.3	3	18.8	2	33.3

## Discussion

In this observational pilot study, we undertook a preliminary assessment of the effectiveness of creative arts activities in children and adolescents with MDs in a clinical setting with respect to short-term behavioral and stress-related physiological changes. Specifically, we anticipated improvements in psychobiological outcomes across the different arts activities. Indeed, results suggest partial confirmation of these hypotheses, as will be discussed here.

In line with H1, beneficial psychological effects were observed in the textile design intervention in terms of heightened *alertness*, while both choir singing and textile design interventions showed a tendency toward a positive change in *mood*. Singing led to a significant increase in PedsQL scores, indicating improved HRQL after 1 week. No further significant effects on behavioral outcomes were noted. In support of H2, sCort levels decreased over the 5 days across arts interventions, indicating reduced physiological stress. However, no other significant changes were observed in sIgA levels for any of the four interventions.

Our findings extend previous work by showing that arts interventions may have short-term positive psychophysiological benefits in this target group. In particular, the observation of overall reduced stress is significant, as it plays a critical role in young people’s agency, resilience, and mental health [7, 8]. Implementing stress-reducing interventions in clinical and school settings can most likely help to alleviate psychiatric symptoms and enhance coping abilities. However, this hypothesis needs to be further evaluated within a framework that allows for longer intervention intervals and also more opportunity for the patients to explore different arts activities.

Interestingly, our investigation yielded few changes in the psychological measures of mood states. This contrasts with evidence from previous research in other populations, which demonstrated an improvement in mood alongside a decrease in cortisol values in response to, e.g., group singing (e.g., [17]). Notably, our previous pilot study on choir singing and music listening in children and adolescents with MDs [18], as well as another study involving adolescent women with depression disorders outside a clinical setting [36], also demonstrated significantly decreased sCort levels that were uncorrelated with psychological changes. Such discrepancies could be attributed to the influence of rater and recall biases present in the self-reported psychological outcomes. Furthermore, it has been suggested that this inconsistency may have occurred because biological changes are more quickly observable than self-rated changes. Therefore, it was recommended that behavior should be observed second-by-second, and mood states should be assessed using additional measures [18, 36].

Against our expectation, the singing intervention did not yield significant pre–post effects on psycho-biological measures. This contrasts with earlier clinical and naturalistic studies that have reported favorable influences of musical engagement (choir singing, music listening) on behavioral and physiological measures including cortisol levels in adults [16, 17]. In our previous pilot study involving young people with MDs [18], we did observe a cortisol reduction after 45 min of choir singing, whereas the current intervention lasted for 90 min. Further studies are needed to investigate the stress-reducing potential of singing with a focus on duration and other contextual factors.

No significant changes in sIgA were observed in our study. Consistent with previous research, conflicting results have emerged concerning the relationship between, e.g., music-related activities and immune responses [17]. Currently, our understanding of immune activity during pleasurable activities, such as arts activities, remains limited. Given that this is a preliminary study, we are unable to offer a conclusion based on our findings.

### Limitations

Our study did not employ a randomization procedure. While we fell short of achieving the intended sample size of 20 patients per group, the data collected during our recruitment process can still hold value in guiding the strategy for recruiting participants in a future, larger-scale study.

Future studies will need to establish a control group to assess experimental effects and to facilitate interpretation of the intervention effects. Additionally, it remains uncertain whether the positive biological effects are subject to specific or nonspecific components of the arts interventions as employed in this study. Furthermore, as the psychological ratings were retrospective, collected from patients themselves, it is possible that rater characteristics or recall bias influenced the outcomes increases.

Since the arts program leaders were unable to be blinded to the intervention, the potential for leader bias must be acknowledged. Additionally, it is essential to recognize the challenges in drawing definitive conclusions regarding the effects of arts activities given the limitations such as small sample size and considerable variability among the biological and psychological results.

The data do not allow us to test which aspects of arts activity were most beneficial for biological and psychological outcomes. Engagement in an arts intervention may be beneficial because of increased attention and support from the arts activity leader, doctor, or parents, as well as other patient support, social activation, or a specific benefit of the artistic activity itself.

Beyond these limitations, there remain major methodological challenges in realizing a long-term, controlled, randomized, comparative study with correspondingly large patient numbers in a clinical setting. These challenges were due to the changing nature of the symptoms and to daily mood fluctuations among the participants. Further longitudinal research with larger patient numbers and additional measurements is needed to clarify the heterogeneity of the data and to determine whether the effects remain over a long term, whether they have an impact on the recovery process, and whether they depend on the clinical picture.

## Data Availability

The data that support the findings of this study are available from the corresponding author, [KGH], upon reasonable request.
